# Circulating Tumor DNA Analyses as a Potential Marker of Recurrence and Effectiveness of Adjuvant Chemotherapy for Resected Non-Small-Cell Lung Cancer

**DOI:** 10.3389/fonc.2020.595650

**Published:** 2021-02-15

**Authors:** Peng-Peng Kuang, Ning Li, Zui Liu, Tian-Yu Sun, Shu-Quan Wang, Jia Hu, Wei Ou, Si-Yu Wang

**Affiliations:** ^1^Department of Thoracic Surgery, Sun Yat-sen University Cancer Center, State Key Laboratory of Oncology in South China, Collaborative Innovation Center for Cancer Medicine, Guangzhou, China; ^2^Otorhinolaryngology Hospital, The First Affiliated Hospital, Sun Yat-sen University, Guangzhou, China; ^3^Department of Breast Oncology, Sun Yat-Sen University Cancer Center, State Key Laboratory of Oncology in South China, Collaborative Innovation Center for Cancer Medicine, Guangzhou, China; ^4^Division of Cardiac Surgery, Heart Center, the First Affiliated Hospital of Sun Yat-sen University, Guangzhou, China

**Keywords:** non-small-cell lung cancer, circulating tumor DNA, chemotherapy, recurrence, prognostic

## Abstract

**Background:**

Although adjuvant chemotherapy is established for patients with non-small-cell lung cancer (NSCLC), the long-term survival remains to be improved. Postsurgical circulating tumor DNA (ctDNA) analysis of resectable NSCLC may identify patients at high risk of recurrence after adjuvant chemotherapy and facilitate personalized therapy.

**Methods:**

This analysis included 38 patients who underwent curative-intent resection and received adjuvant chemotherapy for NSCLC. ctDNA analyses of tumor tissue, and pre- and post-operative plasma samples were performed with next-generation sequencing targeting 425 cancer-relevant genes. We define a ctDNA positive event as at least one shared mutation identified simultaneously in the plasma and tumor specimens. The primary endpoint was recurrence-free survival (RFS).

**Results:**

At least one somatic mutation was identified in the tumor tissue of all 38 patients. Tumor tissue-specific mutated ctDNA was detected in the preoperative plasma samples of 19 (50%) patients. ctDNA in preoperative plasma was in good accordance with that in tissue. ctDNA was detectable in the first post-operative pre-chemotherapy samples of 8 of 35 (22.9%) patients and was associated with inferior RFS (HR, 3.69; P = 0.033). ctDNA was detected in the first post-chemotherapy samples of 8 of 36 (22.2%) patients and was also associated with inferior RFS (HR, 8.76; P < 0.001).

**Conclusions:**

Postoperative and post-chemotherapy ctDNA is a promising prognostic marker for resected NSCLC. ctDNA analyses may define a subgroup that remains at high risk of relapse despite standard adjuvant chemotherapy, and may help to inform intensified therapeutic strategies.

## Introduction

Lung cancer remains the most frequently diagnosed cancer and the leading cause of cancer mortality worldwide (1). Non-small-cell lung cancer (NSCLC) accounts for 80% to 85% of total lung cancer cases (2), and surgical resection is the preferred treatment option for patients with locoregionally confined NSCLC (3–6). Although adjuvant platinum-based chemotherapy is established for resected NSCLC, providing a 5% overall absolute survival improvement at 5 years, the long-term survival outcome remains to be improved (7, 8). Currently, the optimal surveillance protocol for patients with NSCLC who received resection and adjuvant therapy remains to be determined. Therefore, a sensitive and specific biomarker the detection of which identifies patients at high risk of relapse after completion of standard therapy and enables early intervention and personalized cancer therapy is a significant unmet need.

The development of noninvasive liquid biopsy methods represents a promising strategy for dynamically monitoring tumor (9). Circulating tumor DNA (ctDNA) can often be detected in the blood circulation and has emerged as an ideal biomarker for monitoring a tumor throughout treatment (10). Studies have shown that detectable ctDNA following surgical resection for lung cancer (11–13), colorectal cancer (14–17), breast cancer (18, 19), melanoma (20), and pancreatic cancer (21), is associated with a high risk of recurrence. In colon cancer, post-chemotherapy detectable ctDNA detection may identify a patient subgroup that remains at high risk of relapse (22). However, to our knowledge, no studies have examined the significance of post-chemotherapy ctDNA analysis in NSCLC.

Herein we report results from a prospective explorative biomarker study in patients with resectable NSCLC who underwent curative resection and received adjuvant chemotherapy. Using next-generation sequencing (NGS) method with a 425 cancer-related genes panel, we aimed to determine whether postsurgical and post-chemotherapy ctDNA analysis could identify patients at a high risk of recurrence after surgery and guide individualized interventions such as targeted therapy or immunotherapy.

## Materials and Methods

### Patients and Samples

The samples analyzed in this study were collected as part of a registered study evaluating the value of ctDNA on resectable NSCLC (GASTO 1035, Clinical trial information: NCT03465241). Patients with operable NSCLC were recruited from June 2016 to February 2019 at Sun Yat-sen University Cancer Center into the GASTO 1035 study. Written informed consent was obtained from all patients. Key inclusion criteria for this study included a diagnosis of histopathologically confirmed resectable stage IB-III NSCLC; no metastasis evident on computed tomography (CT) of the chest and abdomen, and magnetic resonance imaging (MRI) of the brain before surgery; no history of previous anticancer therapy for lung cancer; and having a treatment plan for postoperative adjuvant chemotherapy. The patient’s disease stage and resectability were determined by a medical team, including thoracic surgeons, medical oncologists, and radiologists. Patients were excluded for having another malignancy within the last 5 years. All treatments were administered as per standard of care.

Tumor tissues were collected at surgery. Blood samples for ctDNA analyses were collected before surgery (up to 7 days pre-operatively), postoperatively within 2 weeks (before postoperative adjuvant chemotherapy), after chemotherapy (within a week of the final cycle of chemotherapy) and every 3 to 6 months for up to 3 years. Follow-up included a 3-monthly clinical review during the first 2 years and a 6-monthly review thereafter. Six-monthly CT of the thoracic and upper abdomen and annual brain MRI were scheduled for evaluation of tumor relapse/metastasis by investigators, and further investigation was carried out if clinically indicated. Clinical information was collected from the electronic medical record system. The pathological stage was based on the seventh lung cancer TNM staging system. Clinicians and patients were blinded to ctDNA data. The study was approved by the Ethics Committee of the Guangdong Association Study of Thoracic Oncology (GASTO) and followed the Declaration of Helsinki and Good Clinical Practice Guidelines.

### ctDNA Analysis

Tumor tissues or plasma DNA from 38 eligible patients was performed with next-generation sequencing (Nanjing Shihe Jiyin Biotechnology Inc. Nanjing, China) targeting 425 cancer-relevant genes. For further details, see **Supplementary Methods**. For an individual patient, we define a ctDNA positive event as at least one shared mutation identified simultaneously in the plasma and tumor specimens. Otherwise, it will be defined as ctDNA negative.

### Statistical Analysis

The primary outcome measure was recurrence-free survival (RFS) assessed by standard radiologic criteria. RFS was measured from the date of surgery to the verified first radiologic recurrence/metastasis (local or distant) or death due to NSCLC. Survival analysis was performed by the Kaplan-Meier method. Cox proportional hazards regression analysis was used to assess the association of ctDNA with RFS. All P values were based on two-sided testing, and differences were considered significant at P<0.05. Statistical analyses were carried out with GraphPad Prism (version 8.0.1, GraphPad Software Inc.) and R 3.5.3 (http://www.r-project.org/).

## Results

### Patient Characteristics

**Figure 1:**
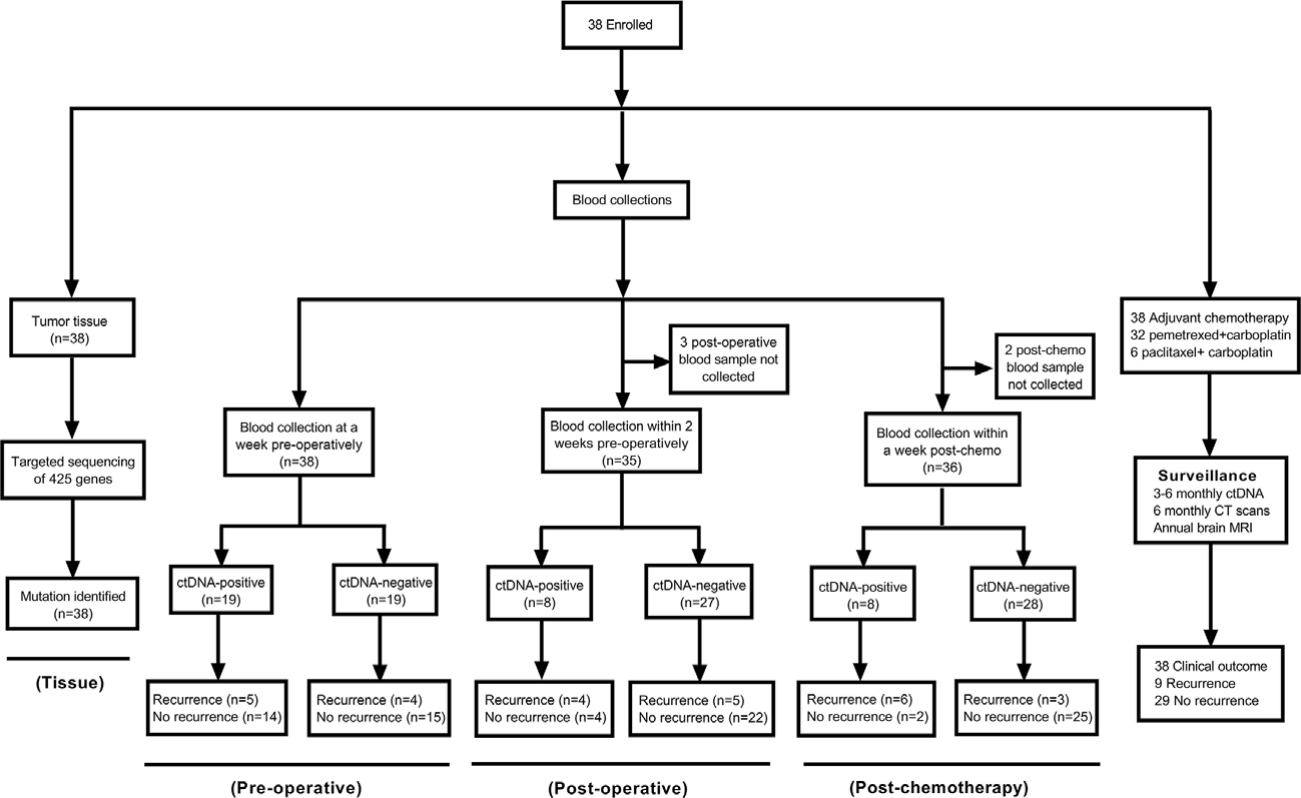
Patient enrollment, sample collections, follow-up, and outcomes.

A total of 38 patients with localized NSCLC (mean [SD] age, 57.4 [7.4] years; 22 [57.9%] male) were included in this analysis. Patient enrollment and the study design are shown in **Figure 1**. The median follow-up time of the patients was 15.8 months (range, 3.7–36.7 months). The 38 patients included 7 with stage IB, 16 with stage II, and 15 with stage III. Of note, four patients were staged as pathological IIIB (pT4N2M0) disease post-operatively. Patient characteristics and demographics are shown in **Table 1**. Lobectomy was performed in all but one patient who underwent left pneumonectomy. Mediastinal Lymph Node Dissection was performed for all patients. Most patients (81.6%) received four cycles of pemetrexed and carboplatin, and six patients (15.8%) received four cycles of paclitaxel and carboplatin. Detailed information about chemotherapy is listed in **Supplementary Table 1**.

**Table 1:** Patient characteristic and demographics of eligible patients (N = 38).

Variable	N (%) or median (range)
Age (years)	57.50 (41–71)
Gender	
Female	16 (42.1)
Male	22 (57.9)
Smoking status	
Never	21 (55.3)
Former	17 (44.7)
Histological type	
Adenocarcinoma	23 (60.5)
Squamous cell carcinoma	6 (15.8)
Others	9 (23.7)
Pathological TNM stage	
IB	7 (18.4)
II	16 (42.1)
III	15 (39.5)
Pathological T stage	
T1–2	28 (73.7)
T3–4	10 (26.3)
Pathological N stage	
N0	16 (42.1)
N1	10 (26.3)
N2	12 (31.6)
Tumor differentiation	
Poor	24 (63.2)
Well moderate	14 (36.8)
Tumor location	
Left-sided	15 (39.5)
Right-sided	23 (60.5)

TNM, tumor node metastasis.

### Primary Tumor Analysis and Preoperative Detection of ctDNA

**Figure 2:**
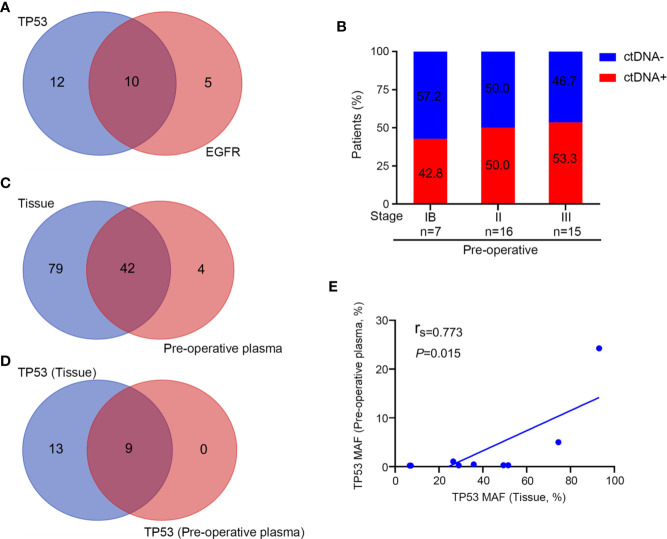
NGS analysis of tissue and preoperative plasma samples. **(A)** *TP53* and *EGFR* mutation detections in tissue samples. **(B)** Preoperative detection of ctDNA in different disease stages. **(C)** The number of detected mutations in tissue and preoperative plasma samples. **(D)** *TP53* mutation detections in tissue and preoperative plasma samples. **(E)** The association of the mutant allele fraction (MAF) in tumor tissue samples and preoperative plasma samples from patients in whom *TP53* mutations were detected in both tissue and plasma samples (n = 9).

For NGS sequencing, the mean coverage depth was 143X for the whole blood control samples, and 1200X for tumor tissues. For ctDNA samples, the mean coverage sequencing depth was 4000X. A summary of frequently mutated genes and mutation frequency is shown in **Supplementary Figure 1**. We observed that *TP53* and *EGFR* were the two most frequently altered genes in the tissues. *TP53* and *EGFR* were observed in tissues of 22 (58%) and 15 (39%) patients, respectively. Ten patients had a comutation of *TP53* and *EGFR* in this cohort (**Figure 2A**). According to the allelic mutation frequency, the next three genes with high-frequency mutations were *GNAS* (n = 6), *RB1* (n = 6), and *PIK3CA* (n = 5). We then analyzed altered genes according to the pathological stage. The number of altered genes detected in individual tumor tissues at different stages was different. Patients in stage I had a numerically low number of mutated genes compared with stage II or III (median 3, 6, and 8 for stage IB, II, and III; **Supplementary Figure 2A**), although the difference is not statistically significant (P = 0.164). Similarly, patients who did not relapse have numerically fewer mutations detected in the tumor tissue (median, 5 vs 11; *P* = 0.068; **Supplementary Figure 2B**).

In the pre-operative context, 19 of these 38 patients (50%) harbored tumor tissue-specific mutation in their plasma (ctDNA-positive), with a ctDNA-positive rate of 42.8% for stage IB, 50% for stage II, and 53.3% for stage III disease. We observed that the detection rate increased along with the stage (**Figure 2B**). Then, matched pairs of tissue and pre-operative plasma samples from 19 patients were used to evaluate the consistency of detection between the “liquid” and “solid” biopsies. A total of 46 mutated genes were detected in the plasma of patients with ctDNA-positive status before surgery, of which 42 (91.3%) could be detected in tissues (**Figure 2C**). Similarly, *TP53* was the most frequently altered gene (nine cases) in pre-operative plasma, which in all of them could be detected in tissues (**Figure 2D**). MAF of *TP53* in the tissues and pre-operative blood samples of the nine patients was further analyzed, and we found that there is a positive correlation between the MAF in tissues and the MAF in plasma samples (r_s_ = 0.773; *P* = 0.015; **Figure 2E**). Taken together, the molecular alterations detected in plasma provided a good representation of the status in the tumor tissue.

### Prognostic Value of Post-Operative ctDNA

**Figure 3:**
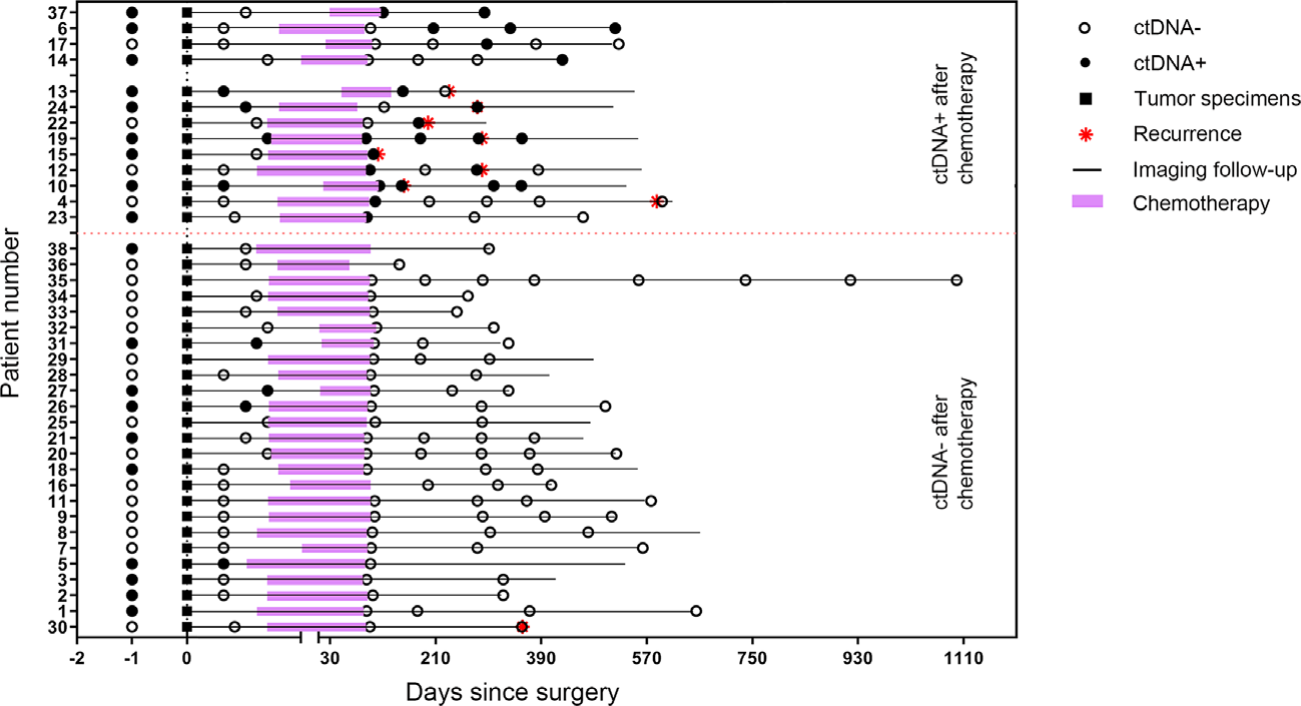
Longitudinal ctDNA profiling results and recurrence status of the 38 patients stratified by post-chemotherapy ctDNA status.

By the time of analysis (December 15, 2019), we observed a recurrence rate of 23.7% (n = 9) among the 38 patients, including six patients treated with pemetrexed and carboplatin and three treated with paclitaxel and carboplatin. The ctDNA results and disease status for all 38 patients are shown in **Figure 3**.

Post-operative plasma specimens were collected within 2 weeks after surgery (before the start of chemotherapy) and were available for 35 patients, of which eight were ctDNA-positive and 27 were ctDNA-negative. Recurrence was detected in four of eight patients (50%) and 5 of 27 patients (18.5%), respectively, suggesting ctDNA-positive patients had a significantly high recurrence rate compared with those who were ctDNA-negative after surgery. Among the eight ctDNA-positive patients, four were stage N2, three were stage N1 and 1 was stage N0. Three of the four patients with stage N2 had postoperative mediastinal lymph node recurrence. One of the three patients with stage N1 also had a recurrence of the mediastinal lymph node. However, 13 patients with stage N0, 6 with stage N1, and 8 with stage N2 disease were ctDNA-negative postoperatively, and only one patient with N1 and one patient with N2 disease had mediastinal lymph node recurrence, respectively (**Supplementary Table 1**).

**Figure 4:**
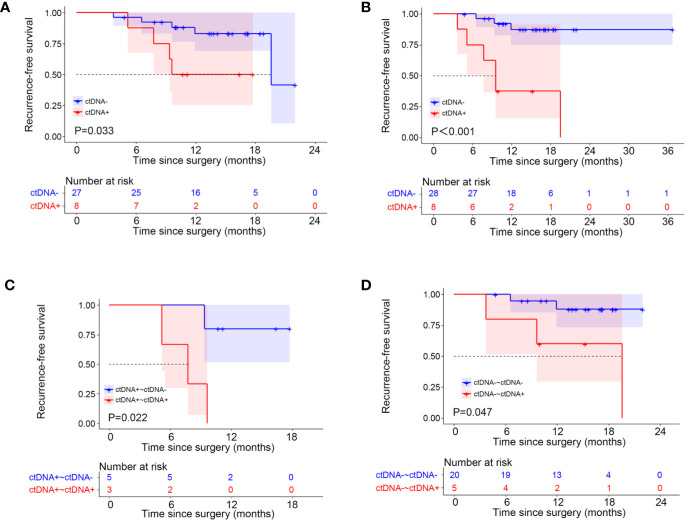
Kaplan-Meier estimates of recurrence-free survival according to circulating tumor DNA (ctDNA) status. **(A)** postoperative ctDNA; **(B)** post-chemotherapy ctDNA; **(C)** postoperative positive ctDNA; **(D)** postoperative negative ctDNA.

Patients with detectable ctDNA in the first post-operative pre-chemotherapy samples had a higher risk of recurrence than those ctDNA-negative patients, with a median RFS of 9.6 months versus 19.6 months (HR = 3.69; P = 0.033; **Figure 4A**).

Besides, we analyzed the changes of ctDNA status before and after surgery in these 35 patients. Of the 10 patients who had ctDNA converted from positive to negative after surgery, only one patient eventually relapsed. However, for the eight patients with detectable ctDNA both pre-operatively and post-operatively, four (50%) patients relapsed. For the 17 patients with undetectable ctDNA both pre-operatively and post-operatively, the recurrence rate is 23.5% (**Supplementary Table 2**). Patients with detectable ctDNA pre-operatively and undetectable ctDNA post-operatively did not have superior RFS to those with detectable ctDNA both pre- and post-operatively, probably due to the small sample size (HR = 5.48; P = 0.086; **Supplementary Figure 3A**). For patients with detectable ctDNA both pre- and post-operatively, the median RFS was 9.6 months. For those with detectable ctDNA pre-operatively and undetectable ctDNA following surgery, the median RFS was not reached (**Supplementary Figure 3A**). Then we compared the survival of patients who had detectable ctDNA pre-operatively and undetectable ctDNA post-operatively (ctDNA+~ctDNA−) with the 17 patients who had undetectable ctDNA both pre-operatively and post-operatively (ctDNA-~ctDNA-), and found that the difference in RFS between the two groups did not differ (P = 0.61; **Supplementary Figure 3B**).

### Prognostic Value of Post-Chemotherapy ctDNA

Overall, first post-chemotherapy plasma was collected within 1 week after the last cycle of chemotherapy and was available for 36 patients. For the 36 patients, nine (25%) experienced a recurrence. We observed a 1-year recurrence rate of 62.5% (five of eight patients) and an overall recurrence rate of 75% (six of eight patients) for patients with a ctDNA-positive finding in the first post-chemotherapy samples. However, only 10.7% of the patients (3 of 28) who were ctDNA-negative in the first post-chemotherapy samples experienced a recurrence during the study period. The positive status of ctDNA in the first post-chemotherapy samples was associated with a shorter RFS, with a median RFS of 9.6 months versus RFS not reached (HR = 8.76; 95%CI, 1.63–47.01; P<0.001; **Figure 4B**).

We then analyzed ctDNA status change before and after completion of chemotherapy to assess the association between ctDNA status and adjuvant chemotherapy (**Supplementary Table 1**). The ctDNA status which changed from positive to negative after completion of adjuvant chemotherapy was noted in five patients (patient nos. 5, 24, 26, 27, and 31). Only one patient of these patients (patient no. 24) eventually recurred. All of the three patients (patient nos. 10, 13, and 19) who did not clear ctDNA experienced disease recurrence, indicating that residual ctDNA is associated with chemotherapy failing to eliminate the residual disease. There were five patients (patient nos. 4, 12, 15, 23, and 37) who had ctDNA converted from negative to positive after chemotherapy. Three of these (patient nos. 4, 12, and 15) patients eventually relapsed. In contrast, for the 20 patients with undetectable ctDNA both pre-chemotherapy and post-chemotherapy, only two of them relapsed, with a recurrence rate of 10% (**Supplementary Table 3**).

For patients with a positive postsurgical ctDNA finding, a positive ctDNA status after chemotherapy was associated with an inferior RFS compared with patients in whom ctDNA became undetectable after chemotherapy (HR = 8.68; P = 0.022; **Figure 4C**). For patients with a negative postsurgical ctDNA finding, a negative ctDNA status after chemotherapy was associated with a superior RFS compared with patients in whom ctDNA became detectable after chemotherapy (HR = 4.76; P = 0.047; **Figure 4D**).

## Discussion

Considerable advances have been made in the development of liquid biopsies in recent years. ctDNA was explored as a sensitive dynamic prognostic or predictive marker of cancer during disease management (9). While the administration of adjuvant chemotherapy is recommended for patients with resectable NSCLC, to date only a 5% overall survival improvement at 5 years has been demonstrated. This study was designed to explore the value of ctDNA analyses in patients with resectable NSCLC undergoing surgery and adjuvant chemotherapy. The results of this study showed that postoperative and post-chemotherapy ctDNA is a useful prognostic marker and might guide personalized adjuvant therapy.

In the study, we define a ctDNA positive event as at least one shared mutation identified simultaneously in the plasma and tumor specimens. We showed that the pre-operative ctDNA-positive rate is 50% in our patients, which is in consistency with previous studies that have reported the ctDNA-positive rate of 48% to 93% (11, 13). The ctDNA-positive rate in our study was highly correlated with tumor stage. The ctDNA-positive rate in pre-surgical plasma was significantly lower in patients with stage I than in those with stage II–III disease. The results are consistent with previous reports reporting the relation of ctDNA and disease stage (23, 24). We observed that TP53 was the most frequently altered gene in both the tissues and plasma.

In our study, the presence of detectable ctDNA in the first post-operation samples was associated with an increased risk of relapse for patients who underwent surgery. This result is in consistency with multiple previous studies conducted in patients with lung cancer (11–13), colorectal cancer (14–17), breast cancer (18, 19), melanoma (20), and pancreatic cancer (21), which have shown that post-operative detectable ctDNA is associated with a significantly increased risk of relapse. Of note, we found that mediastinal lymph node recurrence occurred more often for patients with N2 disease and a positive ctDNA status. These higher-risk patients might be a population where the potential benefits of intensive postoperative therapy (including tyrosine kinase inhibitors, immune checkpoint inhibitors and radiotherapy) could be explored.

Post-chemotherapy analysis of ctDNA appears more informative. Our data have also demonstrated a potential value for ctDNA as a marker of adjuvant therapy benefit. In our study, when ctDNA was detectable after adjuvant chemotherapy, the risk of recurrence was substantially higher than when ctDNA was undetectable. This result in the current study is in keeping with that demonstrated in previous studies in pancreatic cancer (25) and colon cancer (22, 26). Results of the recent study by Tie et al. showed that the 3-year recurrence-free interval was significantly improved for patients of stage III colon cancer whose ctDNA was undetectable compared with those with positive ctDNA findings after chemotherapy (77% vs 30%, P*<*0.001). In another recent study in patients with localized pancreatic cancer, for the 13 post-operative ctDNA-positive patients, doublet chemotherapy was found to show a trend toward improved RFS compared with gemcitabine alone (median, RFSl 10.1 months vs 5.1 months, P = 0.15). These studies suggest that ctDNA analysis can identified a patient subgroup that remains at high risk of relapse despite adjuvant chemotherapy. In our study, of the five patients with positive ctDNA findings pre-operatively and negative ctDNA findings in their first post-chemotherapy samples, only one patient recurred. All the three patients who had positive ctDNA findings both pre-operatively and post-chemotherapy had a disease relapse. Three of five patients whose ctDNA status converted from negative to positive after chemotherapy experienced a recurrence. These results suggest that ctDNA status after chemotherapy is strongly associated with RFS and has the potential to guide intensive postoperative therapy.

Given that a large number of studies have shown that the treatment in patients with a low burden of metastatic disease is far more effective than in patients with a radiologically detectable disease, we believe that patients with detectable ctDNA after chemotherapy may benefit from early intensified treatment. The NGS analysis of pretreatment tissue samples can be used to select therapeutic agents, including gene mutation testing and PD-L1 expression (27, 28). Currently, the prospective clinical trial GASTO1002 that we initiated is ongoing to explore 6-month or 12-month icotinib following chemotherapy to see how well it works compared to chemotherapy alone in treating patients with resected stage IIA-IIIA NSCLC harboring EGFR mutation (NCT01996098). Certainly, immunotherapy as well as other treatment modalities could also be explored. These studies may provide new therapeutic strategies for lung cancer patients who are at high risk of recurrence post-treatment.

The present study has several potential limitations, including the modest sample size, the relatively low event rate, and the absence of a validation cohort. These limitations make the results of our study not conclusive. In addition, we defined the status of ctDNA by comparing the presence of shared mutated genes in plasma and corresponding tissues. This way of defining omits details of specific gene mutations and gene mutation burden. Nevertheless, the results of this study have demonstrated the potential value of post-operative and post-chemotherapy ctDNA analysis in NSCLC. Further well-designed prospective studies are needed to investigate whether ctDNA analysis could be adopted in routine clinical care.

In conclusion, the results of our study provide evidence of the value of postsurgical ctDNA analysis in NSCLC. More importantly, this study highlights the potential clinical utility of postchemotherapy ctDNA analysis in stratifying the risk of disease recurrence. The results of this study may guide personalized cancer treatment by ctDNA analysis and provide a framework for future clinical trials to investigate the clinical benefits of biomarker informed management.

## Data Availability Statement

The datasets presented in this study can be found in online repositories. The names of the repositories and accession numbers can be found below: GSA, Accession HRA000388 (https://bigd.big.ac.cn/gsa-human); Research Data Deposit, RDDA2020001423 (www.researchdata.org.cn).

## Ethics Statement

The studies involving human participants were reviewed and approved by Ethics Committee of the Guangdong Association Study of Thoracic Oncology (GASTO). The patients/participants provided their written informed consent to participate in this study.

## Author Contributions

P-PK, NL, ZL, WO, and S-YW were involved in the design of the study. P-PK, NL, ZL, WO, T-YS, S-QW, and JH were involved in data acquisition. P-PK, NL, ZL, WO, and S-YW were involved in data analysis and interpretation. P-PK, NL, ZL, WO, and S-YW were involved in manuscript preparation. All authors contributed to the article and approved the submitted version.

## Conflict of Interest

The authors declare that the research was conducted in the absence of any commercial or financial relationships that could be constructed as a potential conflict of interest.
