# Supplementary material for: Circulating Tumor DNA Analyses as a Potential Marker of Recurrence and Effectiveness of Adjuvant Chemotherapy for Resected Non-Small-Cell Lung Cancer
Source: Front Oncol. 2021 Feb 15;10:595650. doi: 10.3389/fonc.2020.595650 (PMC7919598; doi:10.3389/fonc.2020.595650)

Supplementary Material

1. Supplementary Methods

Targeted NGS and Sequencing Data Processing

For each patient, Tumor tissue was collected either as fresh frozen (n=35) or as formalin fixed and paraffin embedded tissue (FFPE) (n=3). 5 to 10 mL of peripheral blood was collected from each patient in EDTA-coated tubes (BD Biosciences). Plasma was extracted within 2 hours of blood collection and shipped to the central testing laboratory within 48 hours.

Genomic DNA from FFPE sections or fresh frozen samples and the whole blood control samples was extracted with QIAamp DNA FFPE Tissue kit and DNeasy Blood and tissue kit (Qiagen, USA), respectively. Circulating cell-free DNA (cfDNA) from plasma was extracted with the QIAamp Circulating Nucleic Acid kit (Qiagen). Sequencing libraries were prepared with the KAPA Hyper Prep Kit (KAPA Biosystems) according to manufacturer's instructions for different sample types. Customized xGen lockdown probes (Integrated DNA Technologies) targeting 425 cancer-relevant genes were used for hybridization enrichment. The capture reaction was performed with Dynabeads M-270 (Life Technologies) and xGen Lockdown hybridization and wash kit (Integrated DNA Technologies) according to manufacturers' protocols. Captured libraries were on-beads PCR amplified with Illumina p5 (5' AAT GAT ACG GCG ACC ACC GA 3') and p7 primers (5' CAA GCA GAA GAC GGC ATA CGA GAT 3') in KAPA HiFi HotStart ReadyMix (KAPA Biosystems), followed by purification with Agencourt AMPure XP beads. Libraries were quantified by qPCR with KAPA Library Quantification kit (KAPA Biosystems). Library fragment size was determined by Bioanalyzer 2100 (Agilent Technologies). The target-enriched library was then sequenced on the HiSeq4000 NGS platform(Illumina) according to the manufacturer's instructions. The mean coverage depth was 143X for the whole blood control samples, and 1200X for tumor tissues. For cfDNA samples, the mean coverage sequencing depth was 4000X.

Mutation Calling

Trimmomatic was used for FASTQ file quality control. Leading/trailing low quality (quality reading below 20) or N bases were removed. Paired-end reads were then aligned to the reference human genome (build hg19), with the Burrows-Wheeler Aligner (BWA) with the parameters. PCR deduplication was performed with Picard, and local realignment around indels and base quality score recalibration were performed with GATK3. Matched tumor and normal sample pairs were first checked to have the same SNP fingerprint with VCF2LR (GeneTalk) and nonmatching samples were removed from analysis. Further, samples with mean dedup depth <30X for blood and <600X for plasma were removed. Somatic Single Nucleotide Variant (SNV) calling was performed with Mutect and insertion/deletions (INDELs) were called running Scalpel (scalpel-discovery in –somatic mode). SNVs and INDELs called were further filtered by the following criteria: i) minimum ≥5 variant

supporting reads and ≥1% variant allele frequency (VAF) supporting the variant, ii) filtered if present in > 1% population frequency in the 1000g or ExAC database, iii) filtered through an internally collected list of recurrent sequencing errors (≥3 variant reads and ≤20% VAF in at least 30 out of ~2000 normal samples) on the same sequencing platform. Final list of mutations were annotated with vcf2maf (call VEP for annotation)(1).

Reference:

1. Chalmers ZR, Connelly CF, Fabrizio D, Gay L, Ali SM, Ennis R, et al. Analysis of 100,000 human cancer genomes reveals the landscape of tumor mutational burden. *Genome medicine* (2017) 9(1):34. Epub 2017/04/20. doi: 10.1186/s13073-017-0424-2. PubMed PMID: 28420421; PubMed Central PMCID: PMCPMC5395719.

2. Supplementary Tables

Supplementary Table 2. Preoperative and Postoperative ctDNA status.

Preoperative - Postoperative	N (%)	Recurrence	Recurrence-free	Recurrence rate
Positive-Positive	8 (22.8)	4	4	50%
Positive-Negative	10 (28.6)	1	9	10%
Negative-Positive	0 (0.0)	0	0	0%
Negative-Negative	17 (48.6)	4	13	23.5%

Supplementary Table 3. Pre-chemotherapy and post-chemotherapy ctDNA status.

Pre-chemo - Post-chemo	N (%)	Recurrence	Recurrence-free	Recurrence rate
Positive-Positive	3 (9.1)	3	0	100%
Positive-Negative	5 (15.2)	1	4	20%
Negative-Positive	5 (15.2)	3	2	60%
Negative-Negative	20 (60.6)	2	18	10%

3. Supplementary Figures

Supplementary Figure 1. Summary of frequently mutated genes, mutation frequency in tissue.

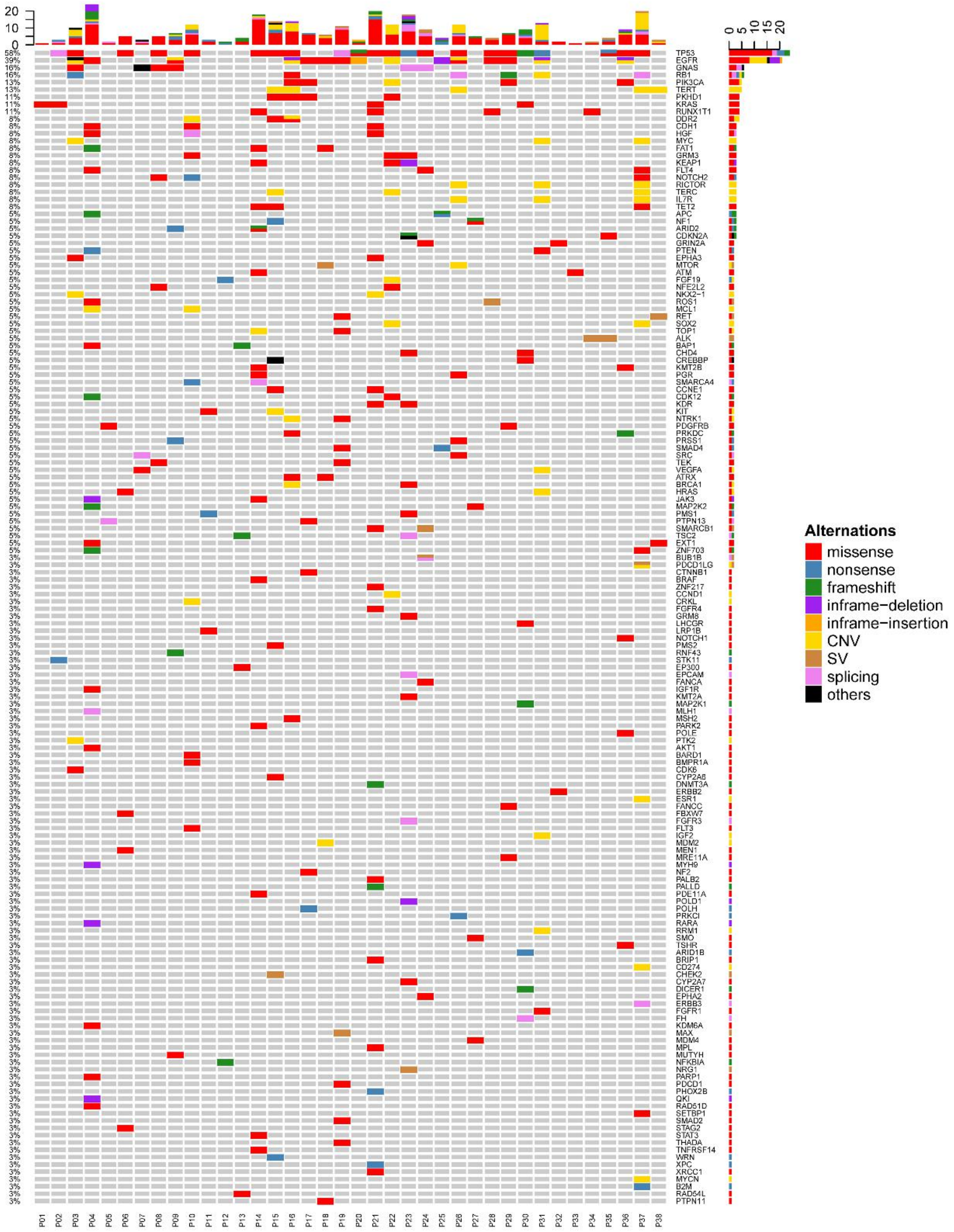

Supplementary Figure 2. The number of detected mutations in tissue. A: IB vs II vs III; **B:** Recurrence vs recurrence-free.

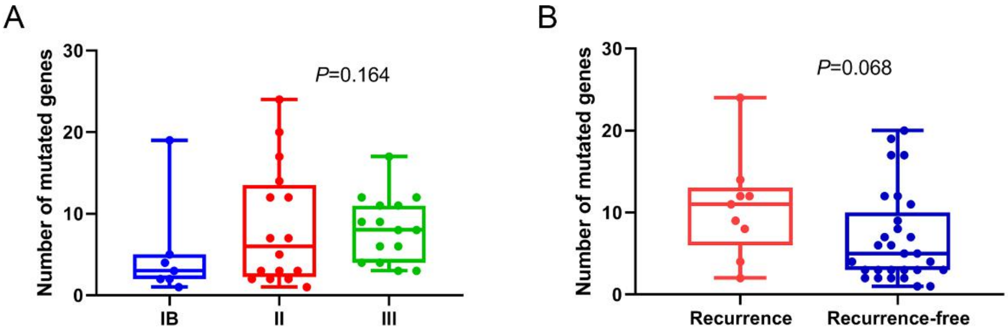

Supplementary Figure 3. Changes in pre-and post-operative ctDNA and RFS. A: ctDNA+~ctDNA- vs ctDNA+~ctDNA+; **B:** ctDNA-~ctDNA- vs ctDNA+~ctDNA-.

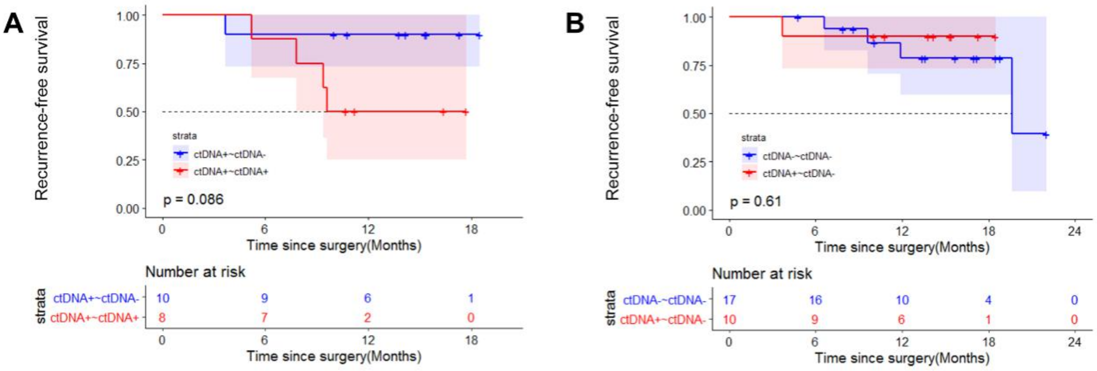

Supplement: Supplementary file 1 [file Presentation_1.zip › Supplementary.pdf]
